# Comment on Jakše et al. Vegan Diets for Children: A Narrative Review of Position Papers Published by Relevant Associations. *Nutrients* 2023, *15*, 4715

**DOI:** 10.3390/nu16111772

**Published:** 2024-06-05

**Authors:** Evgen Benedik, Matjaž Homan, Marko Pokorn, Nada Rotovnik Kozjek, Bernarda Vogrin, Jernej Dolinšek, Matjaž Homšak, Mojca Juričič, Peter Najdenov, Denis Baš, Andreja Širca Čampa, Nataša Bratina, Tadej Battelino

**Affiliations:** 1 University Children’s Hospital, University Medical Centre Ljubljana, 1000 Ljubljana, Slovenia; evgen.benedik@kclj.si (E.B.); matjaz.homan@guest.arnes.si (M.H.); marko.pokorn@kclj.si (M.P.); andreja.campa@kclj.si (A.Š.Č.); natasa.bratina@kclj.si (N.B.); 2 Biotechnical Faculty, University of Ljubljana, 1000 Ljubljana, Slovenia; 3 Faculty of Medicine, University of Ljubljana, 1000 Ljubljana, Slovenia; nkozjek@onko-i.si; 4 Institute of Oncology Ljubljana, 1000 Ljubljana, Slovenia; 5 Outpatient Clinics for Children and School Children Pedenjped, 2230 Lenart, Slovenia; bernarda.vogrin@hotmail.com; 6 Division of Paediatrics, University Medical Centre Maribor, 2000 Maribor, Slovenia; jernej.dolinsek@ukc-mb.si; 7 Slovenian Paediatric Society, 1000 Ljubljana, Slovenia; matjaz_homsak@hotmail.com; 8 Slovenian Association of School, University and Adolescent Medicine, 1000 Ljubljana, Slovenia; juricic.mojca7@gmail.com; 9 General Hospital Jesenice, 4270 Jesenice, Slovenia; peter.najdenov@gmail.com; 10 Paediatric Department at Health Centre Dr. Julija Polca Kamnik, 1240 Kamnik, Slovenia; bas.denis@gmail.com

We have read the recent narrative review article by Jakše et al. on the suitability of a vegan diet for children [1]. We strongly disagree with some aspects of the article and would like to disclose the conflict of interest of the first author and corresponding author of the article.

First, the statements in this article do not represent the professional opinion of the Slovenian Paediatric Society, the Slovenian Association for School, University and Adolescent Medicine, the Slovenian Association for Clinical Nutrition, the Association of Nutritionists and Dietitians and the Slovenian Nutrition Society. Furthermore, some of the statements are not in line with the position paper of the European Society of Paediatrics Gastroenterology, Hepatology and Nutrition [2].

The Professional Expert Panel of Paediatrics, the highest professional body in Slovenia under the Ministry of Health, does not endorse the vegan diet in the paediatric population, as there is no convincing evidence for this type of diet in the most vulnerable population in our society—infants, toddlers, children, adolescents and young adults. In fact, most recent guidelines recommend dietary supplementation in children eating a vegan diet [2,3,4,5,6,7], and two recently published meta-analyses raise concerns about the certainty of the evidence and call for more and better-designed studies given the lack of high-quality data [8,9].

The National Institute of Public Health in Slovenia has also prepared the updated version of the guidelines for healthy eating in educational institutions in Slovenia, which does not support a vegan diet [10]. These guidelines have already been approved by the Professional Expert Panel of Paediatrics and will be implemented in the 2024/25 school year.

Secondly, the first author of the article did not disclose his conflict of interest with Herbalife Nutrition, which has already been disclosed elsewhere [11]: Boštjan Jakše and Barbara Jakše (i.e., Boštjan Jakše’s wife) created the commercial whole-food, plant-based lifestyle program. Part of the supplemental whole-food, plant-based diet uses products from Herbalife Nutrition, from which Boštjan Jakše and Barbara Jakše receive royalty compensation. This clearly indicates a potential conflict of interest that was not disclosed according to *Nutrients’* disclosure policy. Furthermore, the same author claims to be an independent researcher, which is not the case. During his PhD process, the members of the first appointed committee resigned because they considered his research unclear, and, again, he initially failed to disclose a conflict of interest [12].

In addition, the corresponding author of this article, Nataša Fidler Mis, claims to be an employee of the Paediatrics Hospital at the University Medical Centre Ljubljana, Slovenia. She has been appointed by the Prime Minister to chair the newly established Slovenian Strategic Council for Nutrition described elsewhere [13] and has requested that her contract be suspended during this time. Therefore, she is not employed by the Paediatrics Hospital, and her statements in this article do not reflect the position of this institution. Furthermore, she has not disclosed her conflict of interest. Her spouse, Gregor Mis, is the managing director of the advertising company VITA media, which focuses on advertising pharmaceutical and food products. VITA media’s slogan on its website [14] is “The most effective medium when it comes to health”. Again, this is a clear indication of a potential conflict of interest that was not disclosed in accordance with *Nutrients’* disclosure rules.

Consequently, the literature included in the narrative review may lead to a biased view of the vegan diet, particularly in the paediatric population, for whom there is no clear evidence to support this diet [5,6,7,8,9]. New high-quality research in this area in paediatric populations is needed, particularly because of the potential impact of a vegan diet on long-term outcomes related to nutritional programming and effects on the gut microbiota. Together, these can affect the emotional, epigenetic, developmental and cognitive aspects of an individual [15,16,17].

Finally, the authors of the manuscript are members of the National Strategic Council for Nutrition established by the Slovenian government, with most members of the council declaring themselves to be vocal supporters of the vegan diet. Furthermore, it is important to point out that the authors are not paediatricians, and therefore, their knowledge of the possible harmful consequences of an exclusively vegan diet for growing children may be limited. Therefore, this article should be read as a political manifesto rather than a scientific treatise.
